# Association of Inferior Vena Cava Filter Placement With Rates of Pulmonary Embolism in Patients With Cancer and Acute Lower Extremity Deep Venous Thrombosis

**DOI:** 10.1001/jamanetworkopen.2020.11079

**Published:** 2020-07-23

**Authors:** Samyuktha Balabhadra, Joshua D. Kuban, Stephen Lee, Steven Yevich, Zeyad Metwalli, Colin J. McCarthy, Steven Y. Huang, Alda Tam, Sanjay Gupta, Sunil A. Sheth, Rahul A. Sheth

**Affiliations:** 1 Department of Radiology, University of Texas Health McGovern School of Medicine, Houston; 2 Department of Interventional Radiology, University of Texas MD Anderson Cancer Center, Houston; 3 Department of Neurology, UTHealth McGovern School of Medicine, Houston, Texas

## Abstract

**Importance:**

Venous thromboembolism is the second overall leading cause of death for patients with cancer, and there is an approximately 2-fold increase in fatal pulmonary embolism (PE) in patients with cancer. Inferior vena cava (IVC) filters are designed to prevent PE, but defining the appropriate use of IVC filters in patients with cancer remains a substantial unmet clinical need.

**Objective:**

To evaluate the association of IVC filters with the development of PE in patients with cancer and deep venous thrombosis (DVT).

**Design, Setting, and Participants:**

A population-based cohort study was conducted using administrative data on 88 585 patients from the state inpatient databases for California (2005-2011) and Florida (2005-2014). Based on diagnostic and procedure codes, patients with cancer and acute lower extremity DVT were identified. All subsequent hospital visits for these patients were evaluated for the placement of an IVC filter, the development of new PE, the development of new DVT, and in-hospital mortality. Data analysis was performed from September 1 to December 1, 2019.

**Exposures:**

Placement of an IVC filter.

**Main Outcomes and Measures:**

The association of IVC filter placement with rates of new PE and DVT was estimated using a propensity score matching algorithm and competing risk analysis.

**Results:**

The study cohort comprised 88 585 patients (45 074 male; median age, 71.0 years [range, 1.0-104.0 years]) with malignant neoplasms who presented to a health care institution with a diagnosis of acute lower extremity DVT. Of these patients, 33 740 (38.1%) underwent IVC filter placement; patients with risk factors such as upper gastrointestinal bleeding (odds ratio, 1.32; 95% CI, 1.29-1.37), intracranial hemorrhage (odds ratio, 1.21; 95% CI, 1.19-1.24), and coagulopathy (odds ratio, 1.09; 95% CI, 1.08-1.10) were more likely to receive an IVC filter. A total of 4492 patients (5.1%) developed a new PE after their initial DVT diagnosis. There was a significant improvement in PE-free survival for these patients compared with those who did not receive IVC filters across the full, unbalanced study cohort as well as after propensity score matching and competing risk analysis (hazard ratio, 0.69; 95% CI, 0.64-0.75; *P* < .001). Furthermore, IVC filter placement reduced the development of PE in patients with very high-risk malignant neoplasms (eg, pancreaticobiliary cancer), high-risk malignant neoplasms (eg, lung cancer), and low-risk malignant neoplasms (eg, prostate cancer). After accounting for anticoagulation use and imbalanced risk factors, IVC filter placement did not increase the risk of new DVT development.

**Conclusions and Relevance:**

This study suggests that, for patients with cancer and DVT and bleeding risk factors, IVC filter placement is associated with an increased rate of PE-free survival.

## Key Points

**Question:**

Is placement of an inferior vena cava filter associated with a lower rate of pulmonary embolism in patients with cancer and deep venous thrombosis?

**Findings:**

In a population-based cohort study of 88 585 patients with cancer and deep venous thrombosis, the use of inferior vena cava filters was associated with a significant decrease in the rate of pulmonary embolism after accounting for venous thromboembolism risk factors and competing risks.

**Meaning:**

For patients with cancer and deep venous thrombosis, the use of inferior vena cava filters may be warranted.

## Teaser

This cohort study evaluates the association of inferior vena cava (IVC) filters with the development of pulmonary embolism (PE) in patients with cancer and deep venous thrombosis (DVT).

## Introduction

Venous thromboembolism (VTE) is the second overall leading cause of death for patients with cancer.^1^ It is estimated that there is a 4-fold to 7-fold increase in VTE with known malignant neoplasms.^2,3^ One potentially lethal consequence of VTE in patients with cancer is the development of pulmonary embolism (PE). There is an approximately 2-fold increase in fatal PE in patients with cancer.^4^ Although anticoagulation is the standard of care therapy for patients with cancer and deep venous thrombosis (DVT) at risk for PE, many patients are unable to receive anticoagulation because of the risk of bleeding. For these patients, inferior vena cava (IVC) filter placement is frequently performed to reduce the risk of PE.^5,6^

Despite the widespread use of IVC filters, however, the data regarding their effectiveness in reducing the risk of PE are limited. This knowledge gap in IVC filter utility and outcomes is even more pronounced for patients with cancer because very few studies have investigated this patient population, to our knowledge.^7,8^ Defining the appropriate use of IVC filters in patients with cancer remains a substantially unmet clinical need. This need is compounded by the fact that IVC filter placement is not without risk; because the devices themselves are thrombogenic, one of the most commonly reported complications associated with IVC filters is the development of new DVT or propagation of existing DVT.

The purpose of this study was to perform a population-based assessment of the association of IVC filter placement with the prevention of the development of PE in patients with cancer and known DVT. Secondary outcomes included subgroup analyses of outcomes for patients with malignant neoplasms at very high risk, high risk, and low risk of VTE, as well as the association of IVC filter placement with the development of subsequent DVT.

## Methods

### Data Source

This study used encounter-level data provided by the Healthcare Cost and Utilization Project (HCUP). Because these data sets are composed of deidentified medical information, they do not require institutional review board review, in accordance with federal regulations. This study was conducted in compliance with the Report of Studies Conducted Using Observational Routinely Collected Health Data guidelines for administrative claims data. This report follows the Strengthening the Reporting of Observational Studies in Epidemiology (STROBE) reporting guideline for cohort studies.^9^

The HCUP databases are the largest collection of hospital care data in the United States. Furthermore, compared with other private insurance–based databases or Medicare-based data, the HCUP databases include patients across all insurance payors. For this study, we analyzed inpatient hospital encounters through the state inpatient databases for California from 2005 to 2011 and Florida from 2005 to 2014. Because state inpatient database data sets provide encounter-level information, individual anonymized participants can be tracked longitudinally across the entirety of the study duration. California and Florida were selected because of their large populations, demographic and socioeconomic diversity, and geographical distribution.

### Study Population

All patients who presented to a health care institution with a diagnosis of DVT were first identified based on the appropriate *International Classification of Diseases, Ninth Revision* (*ICD-9*) codes (eTable in the Supplement). The diagnosis of DVT was based on the *ICD-9* codes for acute thrombosis of lower extremity deep veins (453.40, 453.41, and 453.42) and for IVC thrombus (453.2). Diagnostic codes for upper extremity thrombosis (453.83), superficial veins (453.6), or chronic lower extremity DVT (453.50-453.52) were excluded. The characterization of acute DVT as a diagnosis present on admission was made by using the HCUP database’s “DXPOA” indicator.

Of the patients found to have acute lower extremity DVT, those with malignant neoplasms were then identified by *ICD-9* codes and comprised the study cohort. These patients were then evaluated longitudinally across the duration of the study period for placement of an IVC filter, development of PE, and development of a new DVT. In addition, data on the demographic characteristics and comorbidities of the patients at the initial presentation for DVT were collected, including age, sex, cancer type, presence of metastases, bleeding risk factors, and coagulopathy. Patients were also evaluated for use of anticoagulation during the study period.

### Study Outcomes

The primary outcome was the development of a PE after the initial DVT diagnosis. The diagnosis of PE was based on *ICD-9* codes for acute PE (415.1), iatrogenic PE (415.11), saddle PE (415.13), and massive PE (415.19); diagnostic codes for chronic PE (416.2 and V12.55) and septic PE (415.12) were excluded. Pulmonary embolism–free survival was measured from the date of the initial DVT diagnosis until the development of PE. If patients received a diagnosis of PE at the same time as their initial DVT diagnosis, they were not considered to have developed a new PE unless they presented again with a new diagnosis of PE at a subsequent hospital encounter. Secondary outcomes included the development of new DVT subsequent to the initial DVT encounter. Patients were considered to have developed a new DVT if they were readmitted with DVT as the principal admission diagnosis at a time subsequent to their initial DVT encounter. Additional secondary outcomes included assessing for the association of IVC filter placement with the development of PE within subsets of patients with archetypal very high-risk, high-risk, and low-risk malignant neoplasms, as defined by the score by Khorana et al.^10^

### Statistical Analysis

All statistical analyses were performed from September 1 to December 1, 2019, using R, version 3.5.1 (R Foundation for Statistical Computing). All *P* values were from 2-sided tests and results were deemed statistically significant at *P* < .05. Univariate analysis of demographic variables and comorbidities was performed using the Fisher exact test, the χ^2^ test, and the Wilcoxon rank sum test, as appropriate. Because large populations tend to overestimate the differences between groups, effect sizes were reported for comparative analysis. Effect sizes and 95% CIs were calculated using the Cohen *d* for continuous variables and the φ coefficient for categorical variables. Multivariable analysis was performed by logistic regression.

Because of the possibility that the patients who underwent IVC filter placement would have greater risk factors and therefore a higher mortality rate, competing risk analysis was performed. The cumulative incidence for PE was calculated from the date of the initial DVT diagnosis to the date of the hospital encounter in which PE was diagnosed. Deaths without PE were treated as competing events. If neither death nor PE occurred, patients were censored at their last hospital encounter. The Gray test was used to determine the association between IVC filter placement and development of PE.

To further account for differences in baseline comorbidities, a propensity score model was constructed using a “nearest neighbor” algorithm.^11^ A 1:1 ratio of patients who received IVC filters and those who did not was used. Candidate variables in the matching algorithm included all variables significantly associated with IVC filter placement via logistic regression, with a threshold of *P* < .10. Furthermore, given the fact that large populations tend to overestimate the differences between groups, additional considerations were taken in variable selection. Specifically, to reduce the risk of selection bias, covariates that were strongly associated with the treatment (ie, IVC filter placement) and outcome (ie, PE) were chosen.^12^ After calculation of propensity scores, the distribution was evaluated using histograms to ensure overlap between patients who did and patients who did not undergo IVC filter placement. The validity of the matching algorithm was confirmed by identifying no statistically significant differences within the matched cohort with regard to baseline covariates. A threshold of 0.1 for standardized mean differences was used to evaluate for balance after propensity matching.^13^ Furthermore, additional propensity matching was performed within subgroups of patients with pancreaticobiliary, gastrointestinal tract, lung, and prostate cancers to evaluate the association of IVC filters across malignant neoplasms with a diversity of VTE risk.

Pulmonary embolism–free survival was assessed using Kaplan-Meier statistics for both unmatched and matched cohorts. Given the possibility that not all patients would receive an IVC filter at the same time as their initial DVT diagnosis, leading to the risk of immortal time bias,^14^ a time-dependent outcomes analysis was performed in the following manner: Cox proportional hazards regression analysis was used to evaluate the association between IVC filter placement and development of PE, treating IVC filter placement as a time-varying exposure.

## Results

Of a total of 332 004 adult patients with hospital encounters for DVT, 88 585 patients (45 074 male; median age, 71.0 years [range, 1.0-104.0 years]) with malignant neoplasms were identified and comprised the study cohort. These patients were analyzed across 534 623 inpatient hospital encounters. The median follow-up time was 479 days (interquartile range, 89-1322 days). A total of 33 740 patients (38.1%) underwent IVC filter placement; most of the IVC filter placement procedures (27 123 of 33 740 [80.4%]) occurred within 30 days of initial DVT diagnosis. Patients with risk factors such as upper gastrointestinal bleeding (odds ratio, 1.32; 95% CI, 1.29-1.37), intracranial hemorrhage (odds ratio, 1.21; 95% CI, 1.19-1.24), and coagulopathy (odds ratio, 1.09; 95% CI, 1.08-1.10) were more likely to receive an IVC filter. The study population’s demographic characteristics and comorbidities are summarized in Table 1.

**Table 1.** Characteristics of Study Cohort

Characteristic	Patients, No. (%)		*P* value	Effect size (95% CI)
	IVC filter (n = 33 740)	No IVC filter (n = 54 845)		
Age, median (range), y	72.0 (2.0-104.0)	71.0 (1.0-103.0)	<.001	0.06 (0.05-0.07)
Female sex	16 363 (48.5)	27 148 (49.5)	.003	0.01 (0.0-0.02)
Race/ethnicity				
White	23 618 (70.0)	39 707 (72.4)	<.001	0.04 (0.03-0.05)
Black	4116 (12.2)	6197 (11.3)		
Hispanic	4791 (14.2)	6745 (12.3)		
Intracranial hemorrhage	978 (2.9)	767 (1.4)	<.001	0.05 (0.05-0.06)
Upper gastrointestinal bleeding	944 (2.8)	383 (0.7)	<.001	0.05 (0.05-0.06)
Lower gastrointestinal bleeding	2631 (7.8)	2303 (4.2)	<.001	0.07 (0.07-0.08)
Hematuria	1889 (5.6)	1755 (3.2)	<.001	0.06 (0.05-0.06)
Proximal DVT	17 882 (53.0)	23 638 (43.1)	<.001	0.1 (0.08-0.11)
Metastatic disease	13 698 (40.6)	18 756 (34.2)	<.001	0.08 (0.07-0.09)
Coagulopathy	4622 (13.7)	5649 (10.3)	<.001	0.05 (0.05-0.06)

Abbreviations: DVT, deep venous thrombosis; IVC, inferior vena cava.

**Figure 1.**
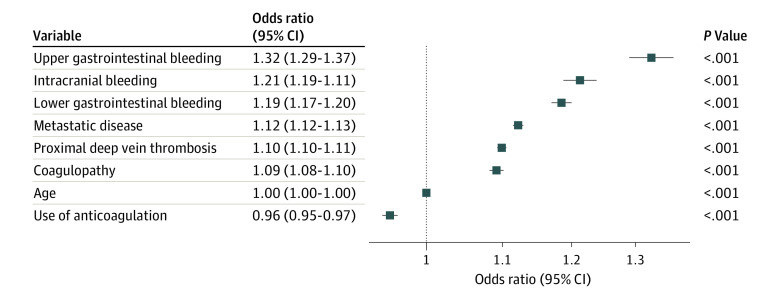
Association of Comorbidities with Inferior Vena Cava (IVC) Filter Placement Multivariable analysis reveals increased likelihood of numerous comorbidities among patients who underwent IVC filter placement compared with those who did not. However, the use of anticoagulation was negatively associated with the placement of IVC filters.

A total of 34 057 patients (38.4%) received systemic anticoagulation during the study period. Anticoagulation use was significantly greater in patients who did not receive an IVC filter compared with those who did (21 773 of 54 845 [39.7%] vs 9784 of 33 740 [29.0%]; *P* < .001). In concordance with this finding, both univariate and multivariate analysis demonstrated that patients who received IVC filters were more likely to have comorbidities that would have precluded the use of anticoagulation at the time of initial DVT diagnosis (Figure 1). The use of anticoagulation was negatively associated with the placement of an IVC filter (odds ratio, 0.95; 95% CI, 0.94-0.97). These covariates were selected for use in the propensity score matching algorithm, described subsequently.

**Figure 2.**
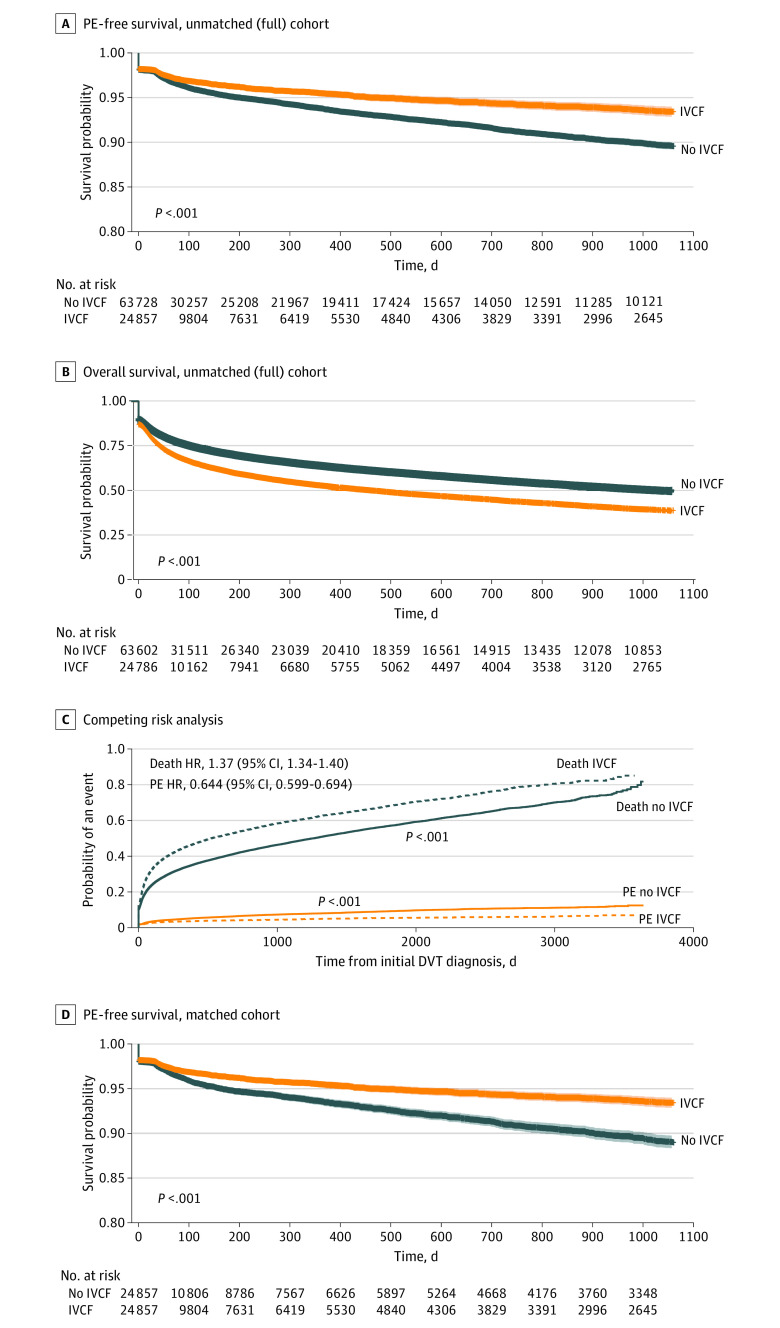
Pulmonary Embolism (PE)–Free Survival and Overall Survival A, Across the entire, unbalanced study cohort, there was a significant improvement in PE-free survival for patients who underwent inferior vena cava filter (IVCF) placement. The shaded panels are 95% CIs. B, There was an increased rate of mortality in patients who underwent IVCF placement. C, There was a persistent improvement in the rates of new PE for patients who underwent IVCF placement after accounting for death as a competing risk. D, The improvements in PE-free survival were also seen after propensity score matching. The shaded panels are 95% CIs. DVT indicates deep venous thrombosis; HR, hazard ratio.

A total of 4492 patients (5.1%) developed a new PE after their initial DVT diagnosis. Most patients presented with a PE within 6 months of their initial DVT diagnosis (eFigure 1 in the Supplement). Despite the increased prevalence of VTE risk factors such as proximal DVT, coagulopathy, and inability to receive anticoagulation in patients who underwent IVC filter placement, there was a significant improvement in PE-free survival across the full, unbalanced study cohort for these patients compared with those who did not receive IVC filters (Figure 2). This finding was true based on time-fixed analysis (Figure 2) as well as time-variable analysis using Cox proportional hazards regression (hazard ratio, 0.69; 95% CI, 0.64-0.75; *P* < .001).

Patients who underwent IVC filter placement had worse overall in-hospital mortality compared with those who did not, reflecting the overall poorer status of patients who underwent IVC filter placement. However, after accounting for mortality as a competing risk, there was a persistent improvement in the rate of PE development for patients who underwent IVC filter placement. Furthermore, after performing propensity score matching, this improvement in PE-free survival remained significant. The matched cohort was comprised of 24 857 patients in the control and 24 857 patients in the treatment cohort (eFigure 2 in the Supplement).

**Figure 3.**
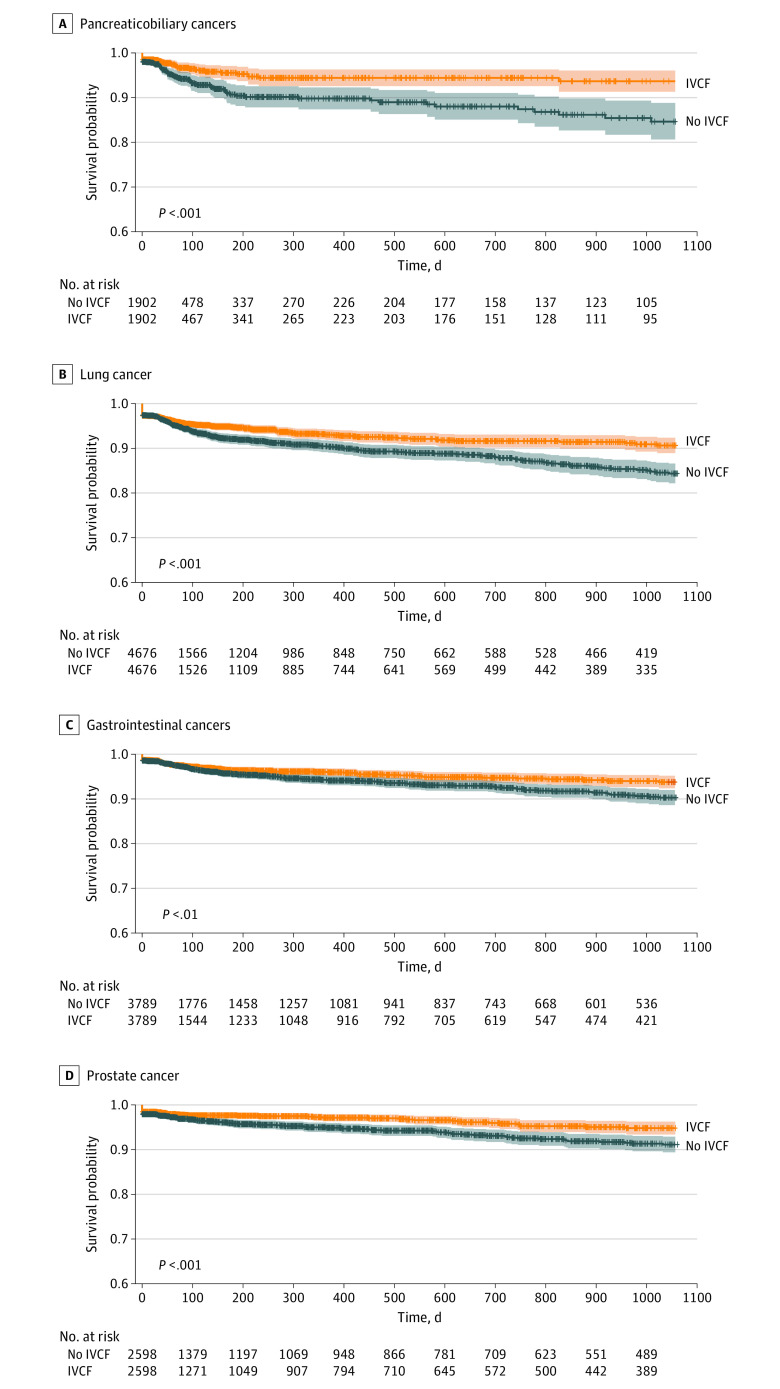
Pulmonary Embolism (PE)–Free Survival Among Patients With Pancreaticobiliary, Gastrointestinal Tract, Lung, or Prostate Cancers Pulmonary embolism–free survival for patients with or without inferior vena cava filters (IVCFs) within subgroups of patients with pancreaticobiliary, gastrointestinal tract, lung, or prostate cancers, matched across covariates found to be significant for IVCF placement. Across this range of histologic characteristics with very high, high, and low risk of venous thromboembolism, IVCF placement significantly decreased the rate of new PE events.

A variety of cancer histologic types were present in the study population (eFigure 3 in the Supplement). The most common cancer was lung cancer (16 085 of 88 585 [18.2%]), followed by gastrointestinal tract cancers (12 668 of 88 585 [14.3%]), hematologic malignant neoplasms (11 162 of 88 585 [12.6%]), and prostate cancer (9213 of 88 585 [10.4%]). The placement of an IVC filter resulted in a significant improvement in the rate of new PE diagnosis for most, but not all, cancer types in this study cohort (Table 2). Placement of an IVC filter was associated with reduced development of PE in patients with malignant neoplasms across the spectrum of VTE risk. That is, IVC filter placement reduced the development of PE in patients with very high-risk malignant neoplasms (eg, pancreaticobiliary), high-risk malignant neoplasms (eg, lung and bladder), and low-risk malignant neoplasms (eg, prostate) (Figure 3).

**Table 2.** Development of PE and Association of IVC Filter Placement Across Cancer Histologic Types in the Study Cohort

Malignant neoplasm	Patients, No. (%)				*P* value
	IVC filter placement (n = 33 740)	Total PE (N = 88 585)	PE in patients with IVC filters (n = 33 740)	PE in patients without IVC filters (n = 54 845)	
Bladder	11 100 (32.9)	3454 (3.9)	877 (2.6)	2468 (4.5)	.01
Brain	16 363 (48.5)	4429 (5.0)	1450 (4.3)	3071 (5.6)	.20
Breast	7827 (23.2)	5137 (5.8)	1180 (3.5)	3564 (6.5)	<.001
Gastrointestinal tract	10 088 (29.9)	4074 (4.6)	1079 (3.2)	2851 (5.2)	<.001
Gynecologic or reproductive	9885 (29.3)	4074 (4.6)	1214 (3.6)	2742 (5.0)	.009
Head or neck	7389 (21.9)	4340 (4.9)	978 (2.9)	3016 (5.5)	.25
Lung	9818 (29.1)	5580 (6.3)	1552 (4.6)	3839 (7.0)	<.001
Hematologic	7861 (23.3)	4252 (4.8)	1180 (3.5)	2851 (5.2)	<.001
Melanoma	11 066 (32.8)	3720 (4.2)	742 (2.2)	2742 (5.0)	.10
Neuroendocrine	7152 (21.2)	4163 (4.7)	3374 (10)	1755 (3.2)	.10
Pancreaticobiliary	9211 (27.3)	3897 (4.4)	910 (2.7)	2797 (5.1)	<.001
Prostate	9480 (28.1)	4252 (4.8)	1012 (3.0)	3016 (5.5)	<.001
Renal	8165 (24.2)	5049 (5.7)	1450 (4.3)	3400 (6.1)	.05
Sarcoma	8435 (25.0)	3809 (4.3)	742 (2.2)	2797 (5.1)	.07
Thyroid	6916 (20.5)	7529 (8.5)	3205 (9.5)	4497 (8.2)	.90

Abbreviations: IVC, inferior vena cava; PE, pulmonary embolism.

A total of 19 426 patients (21.9%) developed a new DVT subsequent to their initial DVT presentation during the study period. The median time interval from initial DVT presentation to new DVT diagnosis was 44 days (interquartile range, 13-238 days). In the full, unbalanced study cohort, the proportion of new DVT diagnoses was significantly higher in the IVC filter patient population than those who did not receive an IVC filter (9623 of 33 740 [28.5%] vs 9803 of 54 845 [17.9%]; *P* < .001). However, after performing propensity score matching to account for differences in bleeding risk factors, coagulopathies, and the use of anticoagulation, the proportion of new DVT diagnoses in the patients who received IVC filters was slightly lower than in patients who did not receive IVC filters (4638 of 24 857 [18.7%] vs 5492 of 24 857 [22.1%]; *P* < .001). Furthermore, in the propensity score model, there was a significant improvement in DVT-free survival in the IVC filter placement group (eFigure 4 in the Supplement).

To further explore the association of IVC filter placement with subsequent DVT development across the spectrum of cancer histologic subtype–associated risk, we performed propensity score matching within subgroups of patients with pancreaticobiliary, gastrointestinal tract, lung, and prostate cancers. As within the matched cohort across all malignant neoplasms, there were slightly lower rates of DVT with IVC filter placement (pancreaticobiliary cancers, 336 of 1902 [17.7%] vs 418 of 1902 [22.0%]; *P* < .001; gastrointestinal tract cancers, 7578 of 3789 [17.0%] vs 769 of 3789 [20.3%]; *P* < .001; lung cancers, 841 of 4676 [18.0%] vs 1000 of 4676 [21.4%]; *P* < .001; prostate cancers, 526 of 2598 [20.2%] vs 600 of 2598 [23.1%]; *P* = .01).

## Discussion

Despite the widespread use of IVC filters, there are limited data regarding the safety and effectiveness of these devices for patients with cancer. One of the only randomized clinical trials to specifically investigate the outcome of adding an IVC filter to pharmacologic therapy in patients with cancer concluded that there was no added benefit. Barginear et al^15^ randomized 64 patients with cancer and DVT (86%) and/or PE (55%) to receive systemic anticoagulation with fondaparinux with or without an IVC filter. Imaging was performed at days 14, 30, and 56 to assess the VTE burden. No patient developed recurrent DVT, although 2 patients, 1 in each group, developed a new PE. Two patients in the IVC group had complications associated with the IVC filter, specifically, thrombosis requiring thrombectomy as well as prolonged bleeding at the venotomy site requiring hospitalization.

Patients with cancer comprised a small fraction of the study cohort in the PREPIC2 (Prévention du Risque d’Embolie Pulmonaire par Interruption Cave 2) trial.^16^ This was a randomized, 2-group study evaluating the effectiveness of anticoagulation vs anticoagulation with IVC filter for preventing PE recurrence in patients with acute PE. Of the 200 patients in the IVC filter group, 33 had cancer; of the 199 patients in the anticoagulation-alone group, 29 had cancer. There was no significant difference in the rate of new PE between the 2 groups (3.0% in anticoagulation plus IVC filter group vs 1.5% in anticoagulation-alone group; *P* = .50), although there was also no significant difference in complications such as DVT or bleeding. No subgroup analysis for the patients with cancer within the 2 groups was performed.

Likewise, in the PREPIC (Prévention du Risque d’Embolie Pulmonaire par Interruption Cave) study, in which patients with proximal DVT were randomized to receive a permanent IVC filter in addition to anticoagulation vs anticoagulation alone, 16% of patients who received an IVC filter and 12% of patients who did not receive an IVC filter were known to have a malignant neoplasm.^17^ In the 8-year follow-up analysis, IVC filter placement reduced the rate of PE but increased the incidence of DVT and did not improve survival.

This scarcity of data is reflected in several consensus guidelines on the use of IVC filters for patients with cancer. The 2016 International Initiative on Thrombosis and Cancer consensus guidelines^18^ relied on the above trials as well as some retrospective studies to base its recommendations on appropriate IVC filter use. The panelists’ consensus was to recommend IVC filter use for patients with known VTE and a contraindication to anticoagulation or for patients with PE recurrence despite receiving optimal anticoagulation therapy. Likewise, the 2019 American Association of Clinical Oncology clinical practice guideline update^19^ for VTE prophylaxis and treatment also emphasized the limited data on the use of IVC filters as well as their potential risks. The expert opinion of this panel was to recommend IVC filters only for patients with acute (eg, within 4 weeks of diagnosis) VTE who have long-term contraindications to anticoagulation or for patients who have progressive VTE despite optimal anticoagulation.

Population-level studies offer an approach to address the limited data. Although clinical trials are often focused on a specific patient population at a single institution or group of institutions, population health analyses incorporate a wide spectrum and large number of patients and providers. Furthermore, patients can be followed longitudinally across a time frame that is typically impractical for prospective trials. Brunson et al^20^ analyzed hospital encounter data for 14 000 patients with cancer and VTE in California across a 5-year period. The primary end point for this study was recurrent PE at 180 days. The authors found no improvement in recurrent PE rate, in distinction to the current study. There are several explanations for this apparent discrepancy. First, the present study followed up with patients until their last hospital encounter; therefore, the follow-up time was greater. Furthermore, the Brunson et al^20^ study censored patients at death as opposed to treating it as a competing risk; because PE can no longer occur after death, the present study’s use of cumulative incidence function accounts for this fact.

As with any intervention, IVC filter placement is not without risk. Although the procedural technique is associated with low morbidity, the devices themselves can lead to complications. These concerns were highlighted in communications by the Food and Drug Administration in 2010 and 2014^21^ owing to the growing appreciation for risks such as device migration, penetration, and embolization. The most common complication associated with IVC filters, however, is an increased incidence of DVT. As the devices themselves represent foreign material within a blood vessel, they are to some extent thrombogenic and can lead to thrombus formation and propagation. In a single-institution, retrospective study of 529 patients with cancer and DVT, Elting et al^22^ found a significantly greater rate of recurrent DVT among the 20% of patients who had IVC filters placed. Almost all of the IVC filters placed in this study were “bird’s nest” filters, a design with the greatest amount of intraluminal metal and one that is rarely used in contemporary practice. As in the present study, almost all patients (89%) in the study by Elting et al^22^ had the IVC filter placement procedure at the time of initial DVT diagnosis.

Placement of an IVC filter has also been associated with increased short-term mortality. Using similar HCUP databases, Turner et al^23^ examined the association of IVC filter placement with mortality within the specific population of patients with VTE and contraindications to anticoagulation; the percentage of patients in this study with cancer was not provided. Of the 126 030 patients in this study, 36.3% underwent IVC filter placement. The authors found an increased risk of 30-day mortality for these patients compared with those who did not receive an IVC filter (hazard ratio, 1.18). Although these data are compelling given the size of the study cohort, it is important to contextualize the findings. In particular, none of the randomized trials nor a recent population health study^20^ has shown an increase in mortality with IVC filter placement; moreover, the relevance of this potential increase in mortality to the population of patients with cancer is unknown.

### Limitations

The authors acknowledge several limitations to the present study. As with any database-driven population health study, accuracy of the data within the database is substantially associated with the quality of the data analysis and conclusions. For example, it is not possible to verify the clinical accuracy of the DVT diagnoses provided by the database, which is created solely using insurance codes. However, previous studies using *ICD-9* codes have identified high predictive values in identifying VTE.^24^ In addition, this study was limited to inpatient encounters, and as such, out-of-hospital events were not included. Furthermore, many important clinical variables are not available in the HCUP databases. For example, covariates such as laboratory test values, coagulation parameters, type of IVC filter deployed, and method of anticoagulation are not available. The inability to include these covariates limits the potential to account for all biases.

## Conclusions

Placement of an IVC filter is a commonly offered treatment option for patients with VTE. In this study of 88 585 patients, IVC filter placement was performed in patients with greater rates of comorbidities as well as overall poorer survival. However, after accounting for death as a competing risk and comorbidities by propensity score matching, IVC filter placement was found to have a significant association with reduced rates of new PE development for patients with cancer and known VTE; this improvement was seen in cancer types across the spectrum of intrinsic, histologic type–associated VTE risk. Moreover, while IVC filter placement was found to be associated with an increased risk of recurrent DVT compared with patients who did not undergo IVC filter placement, this association was inverted after accounting for bleeding risk factors and the use of anticoagulation. These data suggest that IVC filter use in patients with cancer is of potential benefit in appropriately selected patients, and that further investigations into the appropriate use of these devices is warranted.

## Notes

Supplement. **eTable.** *ICD-9* and *CPT* codes **eFigure 1.** Histogram and Density Plot of the Time Intervals From the Initial Hospital Encounter in Which the Diagnosis of Deep Venous Thrombosis (DVT) Was Made to the Subsequent Hospital Encounter in Which the Patient Presented With a New Pulmonary Embolism (PE) **eFigure 2.** A Love Plot of Standardized Mean Differences for Covariates Used in Propensity Score Matching Demonstrates Excellent Balance for All Covariates Following the Matching Algorithm **eFigure 3.** Bar Chart of Cancer Histologies Within the Study Cohort **eFigure 4.** Recurrent DVT-Free Survival Was Improved Rather Than Worsened in the IVC Filter Placement Group
