# Supplementary material for: Association of Inferior Vena Cava Filter Placement With Rates of Pulmonary Embolism in Patients With Cancer and Acute Lower Extremity Deep Venous Thrombosis
Source: JAMA Netw Open. 2020 Jul 23;3(7):e2011079. doi: 10.1001/jamanetworkopen.2020.11079 (PMC7378756; doi:10.1001/jamanetworkopen.2020.11079)
Supplement: Supplement. — eTable. ICD-9 and CPT codes eFigure 1. Histogram and Density Plot of the Time Intervals From the Initial Hospital Encounter in Which the Diagnosis of Deep Venous Thrombosis (DVT) Was Made to the Subsequent Hospital Encounter in Which the Patient Presented With a New Pulmonary Embolism (PE) eFigure 2. A Love Plot of Standardized Mean Differences for Covariates Used in Propensity Score Matching Demonstrates Excellent Balance for All Covariates Following the Matching Algorithm eFigure 3. Bar Chart of Cancer Histologies Within the Study Cohort eFigure 4. Recurrent DVT-Free Survival Was Improved Rather Than Worsened in the IVC Filter Placement Group [file jamanetwopen-3-e2011079-s001.pdf]

Supplementary Online Content

Balabhadra S, Kuban JD, Lee S, et al. Association of inferior vena cava filter placement with rates of pulmonary embolism in patients with cancer and acute lower extremity deep venous thrombosis. *JAMA Netw Open.* 2020;3(7):e2011079. doi:10.1001/jamanetworkopen.2020.11079

eTable. *ICD-9* and *CPT* codes
eFigure 1. Histogram and Density Plot of the Time Intervals From the Initial Hospital Encounter in Which the Diagnosis of Deep Venous Thrombosis (DVT) Was Made to the Subsequent Hospital Encounter in Which the Patient Presented With a New Pulmonary Embolism (PE)
eFigure 2. A Love Plot of Standardized Mean Differences for Covariates Used in Propensity Score Matching Demonstrates Excellent Balance for All Covariates Following the Matching Algorithm
eFigure 3. Bar Chart of Cancer Histologies Within the Study Cohort
eFigure 4. Recurrent DVT-Free Survival Was Improved Rather Than Worsened in the IVC Filter Placement Group

This supplementary material has been provided by the authors to give readers additional information about their work.

© 2020 Balabhadra S et al. *JAMA Network Open.*

eTable. *ICD-9* and *CPT* codes

	***ICD-9* diagnostic code**	***ICD-9* procedure code**	***CPT* code**
**IVCF**			
IVC filter placement		38.7	37620 (before 2012), 37191 (after 2012)
**DVT and PE**			
Pulmonary embolism	415.1, 415.11, 415.13, 415.19		
DVT (acute, lower extremity)	453.2, 453.40, 453.41, 453.42		
**Comorbidities**			
Intracranial hemorrhage	430, 431, 432.x, 852.2, 852.4, 853.0		
Upper GI bleed	531.0, 531.2, 531.4, 531.6, 532.0, 532.2, 532.4, 532.6, 533.0, 533.2, 533.4, 533.6, 534.0, 534.2, 534.4, 534.6, 578.0		
Lower GI bleed	562.02, 562.03, 562.13, 569.3, 569.85, 578.1, 578.9		
Hematuria	599.7		
Proximal DVT	453.41		
Metastatic disease	196.0 – 199.1, 209.70 – 209.75, 209.79, 789.51		
Coagulopathy	286.0 – 286.9, 287.1, 287.3 – 287.5, 649.30 – 649.34, 289.84		
**Cancer types**			
Lung cancer	160.x, 161.x, 162.x, 163.x, 164.x, 165.x		
Breast cancer	174.x, 175.x		
Renal cancers	189.x		
Prostate cancer	185.x		
Gynecologic cancers	179.x, 180.x, 181.x, 182.x, 183.x, 184.x, 187.x		
Bladder cancer	188.x		
Thyroid cancer	193.x, 194.x		
Gastrointestinal tract cancers	150.x, 151.x, 152.x, 153.x, 154.x		
Pancreaticobiliary cancers	155.x, 156.x, 157.x		
Lymphoma/leukemia	200.x, 201.x, 202.x, 203.x, 204.x, 205.x, 206.x, 207.x, 208.x		
Neuroendocrine cancers	209.x		
Melanoma	172.x		
Head/neck cancers	140.x, 141.x, 142.x, 143.x, 144.x, 145.x, 146.x, 147.x, 148.x, 149.x		
Sarcoma	170.x, 171.x, 175.x		
Brain cancers	191.x, 192.x		

© 2020 Balabhadra S et al. *JAMA Network Open.*

eFigure 1. Histogram and Density Plot of the Time Intervals From the Initial Hospital Encounter in Which the Diagnosis of Deep Venous Thrombosis (DVT) Was Made to the Subsequent Hospital Encounter in Which the Patient Presented With a New Pulmonary Embolism (PE)

Most new PE events occurred within the first 90 days following the DVT diagnosis.

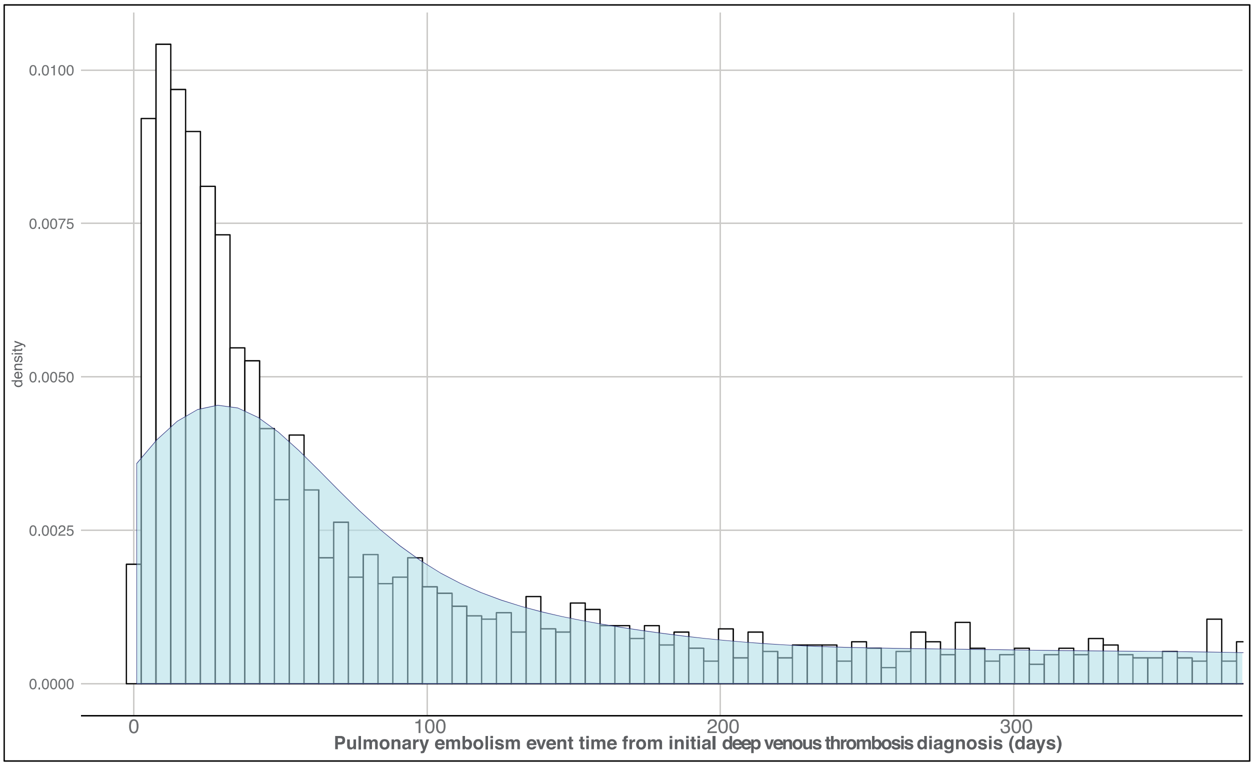

© 2020 Balabhadra S et al. *JAMA Network Open.*

eFigure 2. A Love Plot of Standardized Mean Differences for Covariates Used in Propensity Score Matching Demonstrates Excellent Balance for All Covariates Following the Matching Algorithm

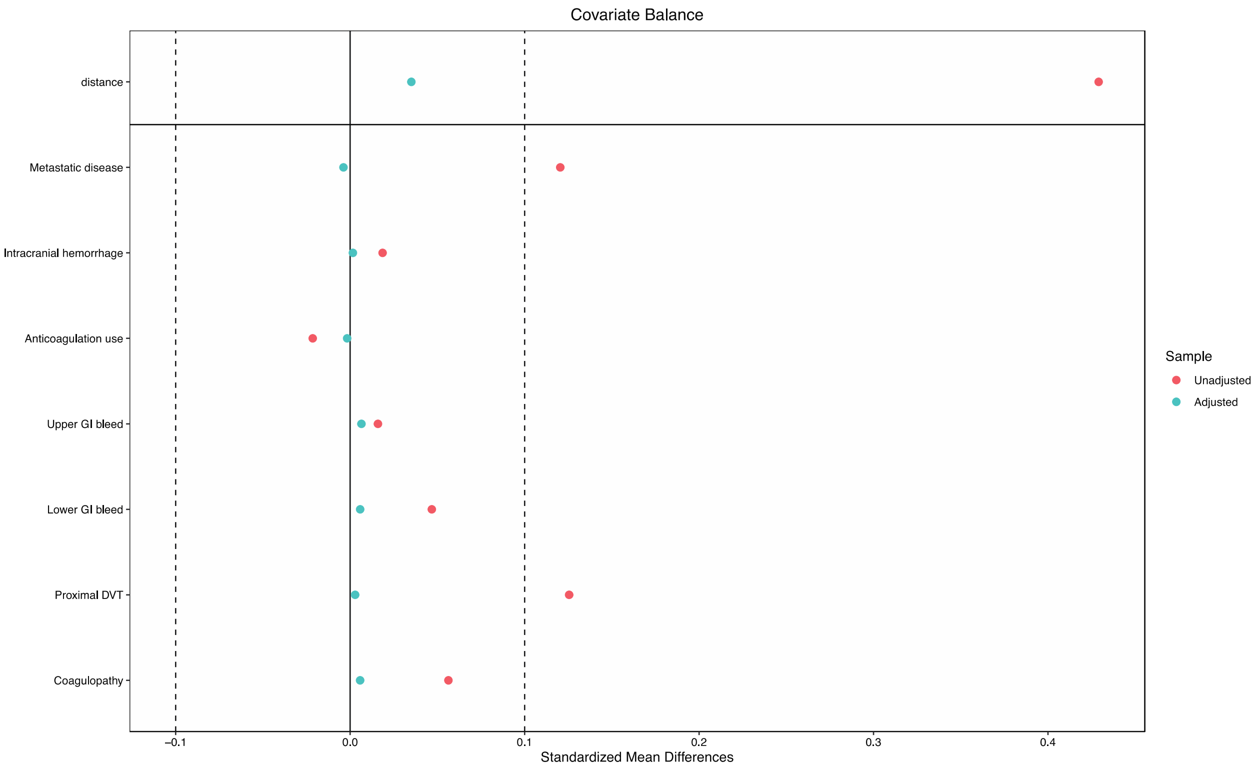

© 2020 Balabhadra S et al. *JAMA Network Open.*

eFigure 3. Bar Chart of Cancer Histologies Within the Study Cohort

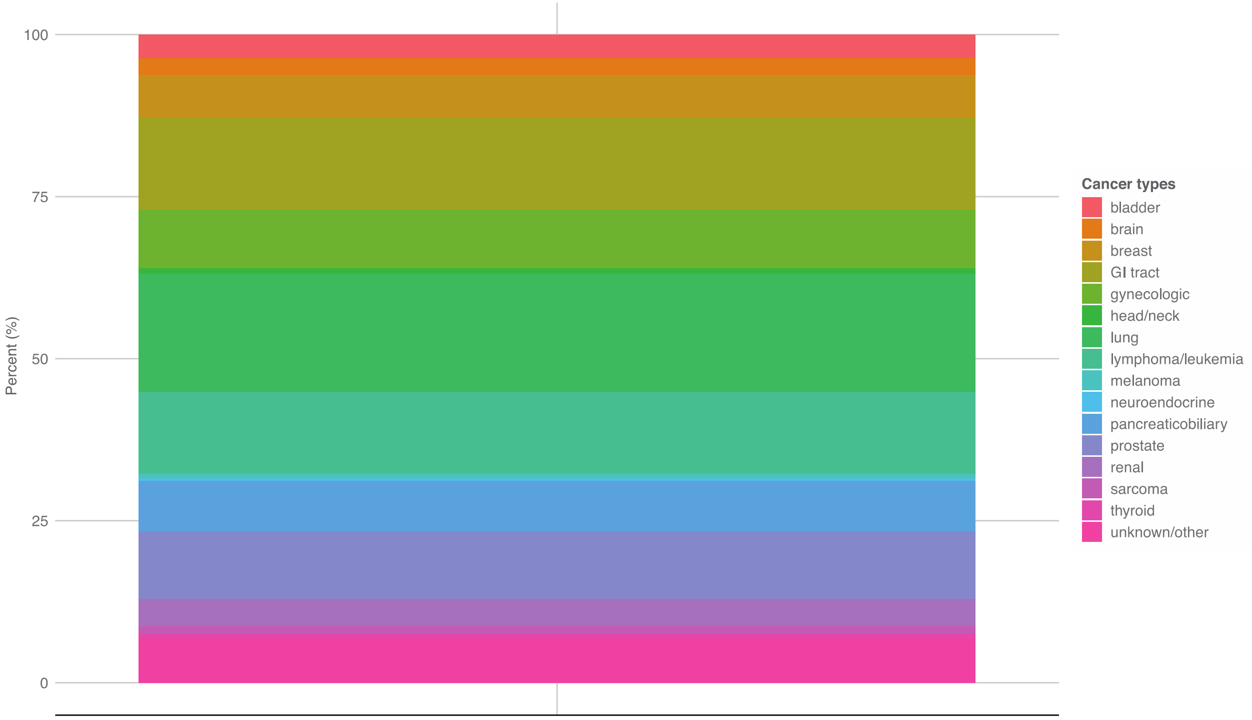

© 2020 Balabhadra S et al. *JAMA Network Open.*

eFigure 4. Recurrent DVT-Free Survival Was Improved Rather Than Worsened in the IVC Filter Placement Group

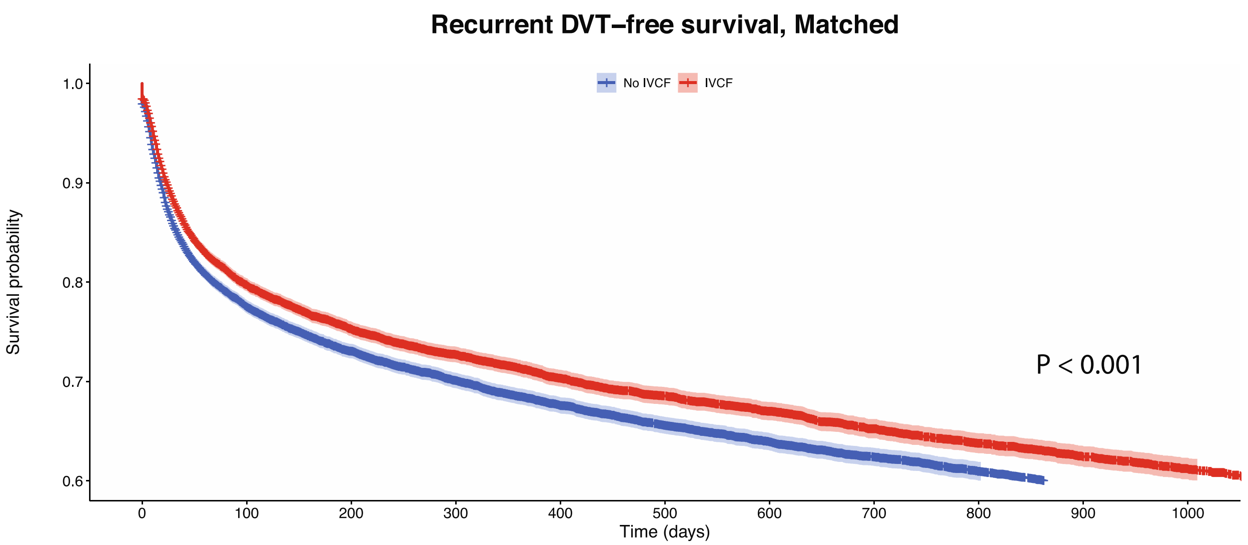

Recurrent DVT–free survival, Matched

	0	100	200	300	400	500	600	700	800	900	1000
No IVCF	24345	9511	7601	6452	5589	4933	4364	3862	3439	3063	2711
IVCF	24470	8749	6656	5539	4718	4099	3603	3176	2785	2451	2161

Time (days)

© 2020 Balabhadra S et al. *JAMA Network Open.*
